# Multi-Modal Multi-Spectral Intravital Macroscopic Imaging of Signaling Dynamics in Real Time during Tumor–Immune Interactions

**DOI:** 10.3390/cells10030489

**Published:** 2021-02-25

**Authors:** Tracy W. Liu, Seth T. Gammon, David Fuentes, David Piwnica-Worms

**Affiliations:** 1 Department of Cancer Systems Imaging, University of Texas MD Anderson Cancer Center, Houston, TX 77030, USA; twliu@mdanderson.org (T.W.L.); stgammon@mdanderson.org (S.T.G.); 2 Department of Imaging Physics, University of Texas MD Anderson Cancer Center, Houston, TX 77030, USA; dtfuentes@mdanderson.org

**Keywords:** intravital imaging, bioluminescence, immune cell imaging, imaging reporters, molecular imaging, tumor signaling, tumor–immune interactions

## Abstract

A major obstacle in studying the interplay between cancer cells and the immune system has been the examination of proposed biological pathways and cell interactions in a dynamic, physiologically relevant system in vivo. Intravital imaging strategies are one of the few molecular imaging techniques that can follow biological processes at cellular resolution over long periods of time in the same individual. Bioluminescence imaging has become a standard preclinical in vivo optical imaging technique with ever-expanding versatility as a result of the development of new emission bioluminescent reporters, advances in genomic techniques, and technical improvements in bioluminescence imaging and processing methods. Herein, we describe an advance of technology with a molecular imaging window chamber platform that combines bioluminescent and fluorescent reporters with intravital macro-imaging techniques and bioluminescence spectral unmixing in real time applied to heterogeneous living systems in vivo for evaluating tumor signaling dynamics and immune cell enzyme activities concurrently.

## 1. Introduction

The tumor microenvironment is well known to contribute to tumor progression [1,2,3,4,5]. However, the tumor immune microenvironment paradoxically can either restrain or promote cancer development and growth [1,2,3,4,5]. Whether tumor–immune and immune–immune interaction networks are anti-tumor or pro-tumorigenic is context dependent, and likely varies during cancer progression, both spatially and temporally. A major obstacle in studying the interplay between cancer cells and the immune system has been the examination of proposed biological pathways and cell interactions in a dynamic, physiologically relevant system in vivo. Conventional molecular and genetic techniques analyze tissue in bulk at different stages of disease development, which only present a glimpse into the molecular changes at a given time; bulk tissue analysis lacks information related to cellular proportions, heterogeneity of genotype, phenotype, and immune cell infiltrates, transient changes at the single cell level, spatial and temporal distribution, or the influence of environmental pressures. Advanced molecular biology and digital genomic techniques (e.g., whole genome sequencing, PCR, reverse phase protein array), or even single-cell RNAseq, mass cytometry, and multiplex imaging may fail to detect the dynamic changes occurring during cancer progression as they are inherently destructive and only provide static analysis or a snapshot in time of the cellular milieu. Thus, to achieve time-course studies using molecular biology techniques, a large number of individual subjects must be included in order to evaluate multiple different time points; moreover, these molecular changes cannot be tracked within the same individual over time and all spatio-temporal correlations are lost. The only method to monitor transiently and spatially encoded changes occurring in an intact in vivo system in real time is through non-invasive imaging.

Bioluminescence imaging has become a standard preclinical in vivo optical imaging technique for tracking cancer cell fates and monitoring tumor growth [6,7,8,9]. The majority of in vivo bioluminescence imaging has been used to track tumor and metastatic burden by stably engineering cancer cells to express constitutively active bioluminescent reporters, which directly measure live cell signaling and metabolism [6,7,8,9]. The versatility of bioluminescence is ever increasing with the development of new emission bioluminescent reporters, advances in genomic techniques, and technical improvements in bioluminescence imaging and processing techniques [6,7,8,9]. Multiplexing using different bioluminescent reporters with various emission spectra allows the study of higher-order molecular and cellular processes in real time in intact living systems simultaneously. Previous reports have demonstrated that luciferases, such as firefly luciferase and click beetle green luciferase, which both use the same D-luciferin substrate, can be imaged and discriminated simultaneously [6,7,8,9,10,11,12,13]. Multi-color bioluminescence can be separated using appropriate emission filters and de-convoluted using spectral unmixing algorithms [13,14]. The theory, software algorithms, and several applications of a new spectral unmixing technique applicable to bioluminescence have been described previously for in vitro applications [10,11,12,13]. In addition, bioluminescent reporters that utilize different substrates are easily resolved in the same animal with separated sequential imaging sessions [6,7,8,9,10,11,12,13,14]. Therefore, different molecular and/or cellular events can be monitored by bioluminescence simultaneously in a single imaging session.

Bioluminescent reporters have been further expanded to study a broad array of biological events. Specific genes can now be studied by bioluminescence when promoter sequences from genes of interest drive luciferase expression or luciferase expression is placed downstream of genetic elements responsive to a desired transcription factor [6,7,8,9,15]. Direct fusion of luciferase to proteins of interest allows the study of protein dynamics and function, including the degradation of signaling proteins, protein–protein interactions, or the regulation of protein stability [6,7,8,9,15]. The combination of different reporters (genetic, protein, and/or cellular) with different luciferases provides a powerful approach to study the temporal and spatial evolution of biological processes in vivo. It is now possible to track cell migration and invasion into surrounding tissues, image specific cell–cell interactions, and monitor molecular changes within a microenvironment using a combination of intravital imaging [16,17,18,19,20,21,22,23,24,25,26] and genetically encoded imaging reporters [6,9,15]. Fluorescence imaging is primarily used with intravital imaging but bioluminescence imaging is also being incorporated, albeit with constitutively active promoters [27,28,29]. Although bioluminescence has several advantages, including ultra-low background signal and high signal-to-noise contrast, it is also limited, similar to other optical techniques, by tissue absorption and scattering of photons [6,7,8]. While intravital imaging techniques require minimally invasive small fields of views, they are one of the few molecular imaging techniques that can follow biological processes at a cellular resolution over long periods of time in the same individual. Herein, we describe an advance of technology with a molecular imaging window chamber platform that combines bioluminescent and fluorescent reporters with intravital macro-imaging techniques and bioluminescence spectral unmixing, demonstrating the imaging of tumor signaling dynamics and enzyme activities in real time in heterogeneous living systems in vivo.

## 2. Materials and Methods

### 2.1. Reagents

Luminol (sodium salt) was purchased from Sigma-Aldrich (Sigma-Aldrich, St. Louis, MO, USA). L-012 sodium salt was purchased from Wako Chemicals USA (Wako Chemicals USA, Inc., Richmond, VA, USA). D-luciferin, potassium salt was purchased from Gold Biotechnology, Inc.**^®^** (Gold Biotechnology, Inc.**^®^**, St. Louis, MO, USA). TNFα was purchased from R&D Systems, Inc. (R&D Systems, Inc., Minneapolis, MN, USA). Luminol sodium salt was dissolved in sterile phosphate-buffered saline (PBS) to a final concentration of 50 mg/mL and stored at −20 °C. Phorbol 12-myristate 13-acetate (PMA) was freshly prepared from a 20 mM stock solution (dissolved in DMSO, stored at −20 °C) and diluted in PBS to a final concentration of 2 mM PMA. L-012 powder was dissolved in sterile double distilled water (ddH_2_0) to a final concentration of 20 mM and stored at −20 °C. Puromycin was purchased from Thermo Fisher Scientific (Thermo Fisher Scientific Inc., Waltham, MA, USA).

### 2.2. Plasmids

The κB5→FLuc plasmid was previously described [30,31]. The FUW-FLG plasmid, encoding a fusion of CBG and EGFP proteins driven by the human ubiquitin C promoter within an established lentiviral backbone, has been previously described [32]. The cytomegalovirus (CMV)-driven pFLuc control plasmid has been previously described [33]. The GFP-IRES-GFP lentivirus was a gift from Ron DePinho. The pDendra2-N and pLVX-IRES-Puro were purchased from Clontech (Clontech, Moutain View, CA, USA). A Cignal lenti NFκB→FLuc reporter was purchased from Qiagen (SABiosceinces, Frederick, MD, USA). 7TFC was a gift from Roel Nusse (Addgene plasmid # 24307; http://n2t.net/addgene:24307 (accessed on 23 February 2010); RRID:Addgene_24307) [34].

### 2.3. Cells

B16F10 melanoma cells were purchased from the MD Anderson Cancer Center Cell core (originally from the American Type Culture Collection (ATCC, Manassas, VA, USA)). B16F10 cells were cultured in DMEM supplemented with 10% heat-inactivated fetal bovine serum (FBS). Pan02 cells were purchased from the DCTD Tumor Repository, National Cancer Institute (Frederick, MD, USA). Pan02 cells were cultured in RPMI 1640 supplemented with 10% heat-inactivated FBS and 2 mM glutamine. Cell cultures were grown at 37 °C in a humidified 5% CO_2_ atmosphere.

### 2.4. Generation of Stable Reporter Cells

Generation of a B16F10 cell line stably expressing GFP: B16F10 cells at 50% confluence were infected with the second generation GFP-IRES-GFP, CBG, or FLuc lentivirus. GFP-IRES-GFP- and CBG-infected B16F10 cells were selected for GFP expression by flow cytometry sorting. FLuc lenti-infected B16F10 cells were selected by bioluminescence imaging; after two weeks, bioluminescence images of isolated cell colonies using phenol-free DMEM media with 10% FBS and 150 µg/mL D-luciferin were acquired to assess reporter gene expression wherein bioluminescent colonies were harvested and expanded. Cells were transduced at a multiplicity of infection (MOI) of 10. Generation of the B16F10 GFP-IRES-GFP cell line stably expressing pκB5→FLuc: cells at 95% confluence were co-transfected with 10 µg of pκB5→FLuc plasmid and 3 µg of pLVX-IRES-Puro plasmid DNA using Fugene6 transfection reagent (Promega, Madison, WI, USA) in a 6-well plate. At 24 h post transfection, media were replaced with fresh cell media, and at 48 h post transfection, cells were trypsinized and plated at a variety of densities (1:2, 1:5, 1:10) into media containing 2 µg/mL puromycin to select for stable transformants. After two weeks, bioluminescence images of isolated cell colonies using phenol-free DMEM media with 10% FBS and 150 µg/mL D-luciferin were acquired to assess reporter gene expression where bioluminescent colonies were harvested and expanded. Cells were continuously cultured in the presence of 2 µg/mL puromycin to maintain expression of the reporter plasmid. Generation of a B16F10 and Pan02 cell line stably expressing Dendra2: cells at 95% confluence were co-transfected with 10 µg of pDendra2-N plasmid using Fugene6 in a 6-well plate. At 24 h post transfection, media were replaced with fresh cell media, and at 48 h post transfection, cells were trypsinized and plated in media containing 1.5 mg/mL G418 for B16F10 cells and 150 µg/mL G418 for Pan02 cells to select for stable transformants. Flow cytometry was used to isolate Dendra2-positive cells; cells were sorted twice for Dendra2 fluorescence (λ_ex_ = 409 nm, λ_em_ = 507 nm). Generation of B16F10 Dendra2 and Pan02 Dendra2 cell lines stably expressing NF-κB→FLuc: B16F10 Dendra2 or Pan02 Dendra2 cells at 50% confluence were transduced with the NF-κB→FLuc lentivirus and selected using 2 µg/mL puromycin. Cells were transduced at an MOI of 10. All cell lines tested negative for mycoplasma.

### 2.5. Animals

The Institutional Animal Care and Use Committee at the University of Texas MD Anderson Cancer Center approved all animal protocols. The following animals were used for window chamber experiments: wild type C57BL/6 animals (Taconic Biosciences, Rensselaer, NY, USA); syngeneic C57BL/6 myeloperoxidase-deficient *MPO^−/−^* (MPO^tm1Lus^, The Jackson Laboratory, Bar Harbor, ME, USA); *p21-FLuc* reporter mice [35]; and *LSL-Kras^G12D/+^;LSL-p53^T172H/+^;Pdx-1-Cre* (*KPC*) animals kindly provided by Dr. Chun Li [36].

### 2.6. Preparation for Window Chamber Implantation Surgery

Animals weighed at least 20 g prior to window chamber implantation surgery. A surgical kit with chlorhexidine, ophthalmic ointment, buprenorphine, and sterile materials was provided by the Department of Veterinary Services at MDACC. Animals were anesthetized using 2% isoflurane in oxygen (2 mL/min flow rate) and the fur was removed from the dorsum. Animals received a single injection of 0.1 mg/kg of buprenorphine prior to beginning the surgery. All surgical procedures were performed in aseptic conditions while maintaining body temperature at physiological levels using a heating pad. The skin was prepared by three alternating washes of chlorhexidine and 70% ethanol and ophthalmic ointment was applied to the animals’ eyes. Before any incisions were made, the toe pinch reflex test was done to determine if the animal had attained a surgical level of anesthesia. 

### 2.7. Dorsal Skin Window Chamber Implantation Surgery

Titanium dorsal skin window chambers were purchased from APJ Trading (APJ Trading Co, Inc, Ventura, CA, USA). The dorsal skin was drawn up into a longitudinal fold using a straight suture needle and sterile silk. The skinfold was trans-illuminated so that the symmetrical pattern of blood vessels on either side of the dorsal midline were matched for an even skinfold. The flat side of one of the window chamber pieces was held against the skin fold and the positions of the screw holes marked. A hole was punched through both sides of the skin at each screw location using an 18 G needle. The front window chamber frame was then screwed into the rear frame and the top of the skinfold sutured along the edge of the frame through the skin to hold the window chamber in place. Antibiotic ointment was applied to all sites where screws and sutures passed through the skin. A 1.2 cm diameter circle in the top layer of skin was cut along the circumference of the window chamber along the frame with scissors. The exposed dermis was washed with saline prior to an injection of 0.5–1 × 106 tumor reporter cells between the exposed fascial plane and dermis. The exposed window area was then filled with saline until a meniscus formed, a glass cover slip was placed over the window and secured with a retaining ring.

### 2.8. Abdominal Pancreas Window Chamber Implantation Surgery

The abdominal window chamber was produced by the UT MD Anderson Cancer Center Radiation Physics machine shop, custom modified from the specifications from Ritsma et al. [37]. After window chamber preparation described above, a sterile drape was placed over the animal with the surgery area remaining uncovered. Using a scalpel, a 15 mm incision through the skin to the dorsal left of the spine was made and the skin retracted. A similar length incision through the muscular layer was then made and the muscular tissue retracted, exposing the abdominal organs. The abdominal window chamber was held in position using a purse-string non-resorbable suture, which was concealed within the groove of the abdominal window chamber ring. A purse-string suture in the muscular layer was first made, followed by a purse-string suture through the skin. Concealing the suture within the groove of the ring prevented mice from biting or pulling the sutures. Once the abdominal window chamber was sutured in place, the pancreas was located and secured by gluing the organ to the interior inner side of the abdominal window chamber using high viscosity cyanoacrylates (Sigma-Aldrich, St. Louis, MO, USA). Once the pancreas was positioned within the abdominal window chamber, an orthotopic injection of 1 × 10^6^ reporter tumor cells occurred (no tumor injection occurred for *KPC* or *KPC-Luc* animals). To further hold the organ in place, an optically translucent silicone (Kwik-Sil™, World Precision Instruments, Sarasota, FL, USA) was placed around the pancreas. A 1.2 cm diameter glass cover slip was then placed on top of the window chamber and held in place by either glue or a retaining ring.

### 2.9. Post Window Chamber Implantation Surgery

After the window chamber implantation, the animal was placed on another heating pad and gently warmed during recovery; animals were injected subcutaneously with 1 mL of saline to maintain hydration and 0.1 mg/kg buprenorphine SR. Animals were monitored until fully recovered from the surgical procedure. Antibiotic ointment was applied along the tissue–implant interface. All animals were housed separately after window chamber implantation and the wire bar lid removed from the microisolator cages. A 2 mg Rimadyl tablet (Bio-Serv, Flemington, NJ, USA) was given orally in hydrogel and placed in the animals’ cages.

### 2.10. In Vivo Imaging

Macroscopic imaging was done using the IVIS Spectrum (Perkin Elmer/Caliper Life Sciences, Waltham, MA, USA). Animals were anesthetized under 2% isoflurane. Acquisition of window chamber bioluminescence images were as follows: acquisition, autoexposure; filters, open, <489 nm, 540/50 nm bandpass, >594 nm and >634 nm; binning, 16; FOV 6.6 cm; f-stop, 2. Custom 60 mm filters designed to fit the IVIS Spectrum were purchased (Semrock, Rochester, NY, USA). The filter specifications were as follows: 492 nm short pass, 540/50 nm band pass, 594 nm long pass, 635 nm long pass with Tav >90%. Imaging of window chamber animals occurred as follows: fluorescence images of tumors if stably expressing fluorophores were imaged (acquisition, autoexposure; reflective illumination, filter, 500 nm excitation, 540 nm emission; binning, 16; FOV, 6.6 cm; f-stop, 2) prior to bioluminescence imaging. Exogenous bioluminescent reporters (luminol and L-012) were administered prior to D-luciferin when both were imaged. Post bioluminescent substrate administration, an imaged was acquired 9 min post each injection. The following administration doses were used for each reporter: luminol (200 mg/kg of body weight, i.p.), L-012 (25 mg/kg of body weight, i.p.), and D-luciferin (150 mg/kg of body weight, i.p.).

### 2.11. In Vivo Spectral Unmixing

In vivo bioluminescence spectra were created using window chamber models where animals were imaged with a single injection of bioluminescent (BLI) reporters. The following administration doses were used for each reporter to generate the in vivo pure spectra: 200 mg/kg of body weight luminol to generate the luminol/L-012 spectrum (luminol and L-012 spectra are similar), and 150 mg/kg of body weight D-luciferin to generate a CBG and a FLuc spectrum. At 9 min post injection, bioluminescence images using all emission filters (<490 nm, 490 nm–590 nm, >594 nm, >635 nm) occurred. Manual unmixing to generate the pure spectra was done using Living Imaging. From each image of the pure reporter, an area of a pure reporter was drawn and a spectrum for the reporter was generated. This spectrum was exported into Excel where the average spectrum was calculated (luminol/L-012, *n* = 3 images; CBG, *n* = 6 images; FLuc, *n* = 6 images). Each individual average spectrum was saved as a csv file (Figure S1E) and imported into the spectrum folder of Living Image. The pure spectrum for each reporter was then available in the Living Image library to be used for spectral unmixing. When spectral unmixing images, only the spectra for reporters that needed to be separated visually were selected.

### 2.12. Window Chamber Quantitative Analysis

Processed spectrally unmixed images of individual reporters were analyzed using the Living Image software (Perkin Elmer/Caliper Life Sciences, Waltham, MA, USA). The spectrally unmixed images of different bioluminescent reporters within the window of individual mice were quantified by drawing a uniform region of interest (ROI) around the 1.2 cm glass cover slip at every imaging time point for each animal. An average background ROI (bkg ROI, remote from the window chamber and animal) was drawn and measured on each spectrally unmixed image. All window chamber quantification of ROIs were background subtracted using the bkg ROI. Changes in signal intensity of were plotted as a function of time. Time-averaged quantification of the mean reporter signal in each animal was then calculated using a step function of signal intensity over the imaging time points. When quantifying the contrast to noise ratio (CNR), an auto-threshold ROI set to 20% was created on each spectrally unmixed BLI image. This ROI was copied onto each BLI image to quantify the CNR. The CNR was calculated using the following equation:

$$\mathrm{CNR}=\;\frac{\left({\mathrm{mean}\;\mathrm{bioluminescence}}_{\mathrm{target}}-{\mathrm{mean}\;\mathrm{bioluminescence}}_{\mathrm{non}-\mathrm{target}}\right)}{{\mathrm{standard}\;\mathrm{deviation}\;\mathrm{of}\;\mathrm{bioluminescence}}_{\mathrm{non}-\mathrm{target}}}$$

In order to also quantify spatial distribution, window chamber spectrally unmixed images were exported to ImageJ. A macro reorienting all the skin window chambers to the same position (using the window chamber screws as reference points) while drawing uniform ROIs around the 1.2 cm glass cover slip was developed in ImageJ and used to align all window chamber images from the same animal (Figure S2, ImageJ code provided in Supplementary Materials). The individual photos corresponding to each reporter were changed to 120 × 120 bicubic, 32-bit images and saved as TIF images using the naming format: S## day # reporter photo. The individual reporter images (unmixed BLI and fluorescence images) for each reporter were saved using the naming format: S## day # reporter. The fluorescence images were changed to 32-bit images prior to saving in the above naming format. A cvs file containing the exposure times for each image was created. Once images were saved and formatted accordingly, the ImageJ macro (Figure S2) was run, resulting in aligned and cropped optical window chamber images. To quantify the NF-κB transcriptional activation normalized to tumor mass, NF-κB→FLuc images were divided pixel by pixel by the Dendra2 fluorescence in ImageJ. A uniform ROI was drawn around the cropped image and the total counts were measured in the NF-κB→FLuc/Dendra2 images for each animal at each time point. Similar to the analysis of Living Image processed images, changes in signal intensity of NF-κB→FLuc/Dendra2 were plotted as a function of time. Time-averaged quantification of mean NF-κB→FLuc/Dendra2 in each animal was then calculated using a step function of signal intensity over the imaging time points. Graphs were made and statistical analyses were performed using GraphPad Prism (GraphPad Software, Inc, La Jolla, CA, USA). Data were expressed as mean ± SEM.

### 2.13. Histological Analysis

Necropsy of animals occurred post 14 days wherein the titanium window chamber was removed, skin excised and tissue frozen in optimal cutting temperature (O.C.T.) embedding media (Thermo Fisher Scientific Inc., Waltham, MA, USA). Prior to histological analysis, tumor samples were formalin fixed for 48 h and stored in 70% ethanol until histological staining. All tumor samples were sent to the Research Histology, Pathology, and Imaging Core at UT MD Anderson Cancer Center where they were sectioned, stained, and analyzed.

### 2.14. Data Availability

All relevant data are within the manuscript and the Supplementary Material files.

## 3. Results

### 3.1. Generation of In Vivo Spectra

**Figure 1:**
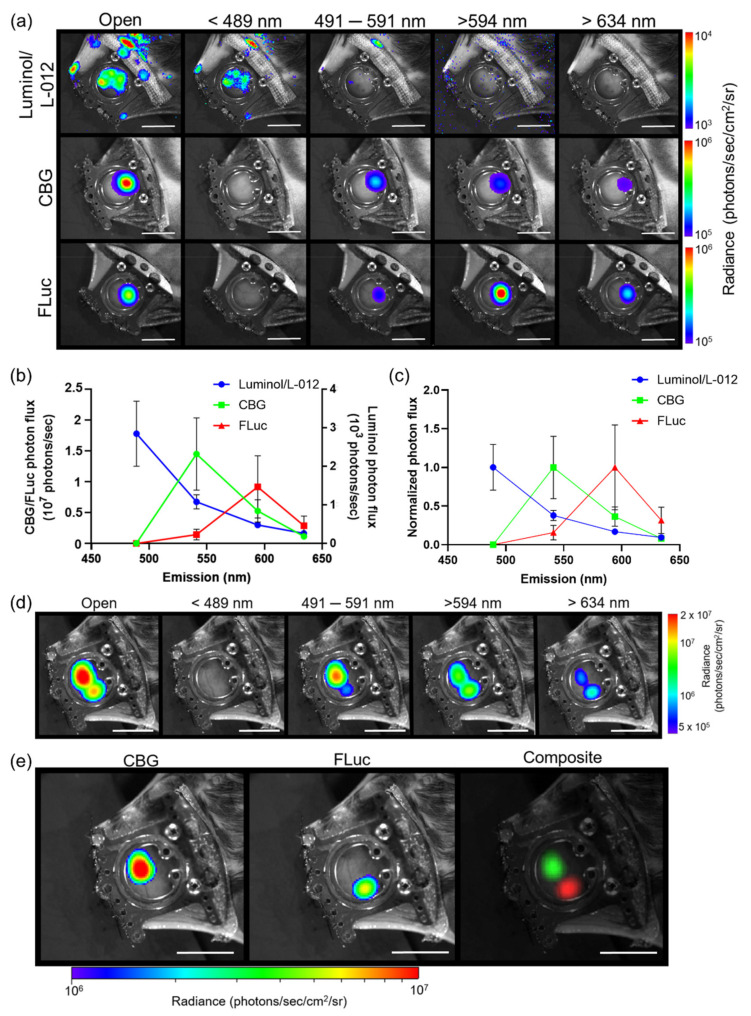
Generation and validation of pure in vivo bioluminescence spectra for spectral unmixing. (**a**) Representative intravital macro-bioluminescence image of separate window chamber-bearing animals for luminol, click beetle green (CBG), and firefly luciferase (FLuc) using different filter settings. (**b**) Mean and (**c**) normalized in vivo emission spectra for different bioluminescent reporters (*n* = 3 animals imaged with luminol, *n* = 6 animals imaged with CBG, *n* = 6 animals imaged with FLuc). In vivo bioluminescence unmixing of B16F10 tumors; one B16F10 tumor stably expressed ubiquitin-CBG (B16F10 CBG) and the other B16F10 tumor stably expressed CMV-FLuc (B16F10 FLuc). (**d**) Representative in vivo bioluminescence image of B16F10 CBG and B16F10 FLuc tumors in the same animal within the same window chamber following a single i.p. injection of D-luciferin using all emission filters. (**e**) Representative unmixed BLI images of B16F10 CBG and B16F10 FLuc tumors in the same window following a single i.p. injection of D-luciferin; two tumors were spectrally unmixed using the CBG FLuc user-generated library. The composite image shows pseudo-colored B16F10 CBG (green) and B16F10 FLuc (red) tumors within the same window chamber (*n* = 2 animals). Scale bar represents 1 cm.

To spectrally unmix different bioluminescent reporters, in vivo pure spectra of each reporter of interest were first generated. In order to achieve this, custom 60 mm emission filters were designed to fit our macro-imaging system. Individual bioluminescence images of three different reporters with various emission spectra were then recorded: luminol (λ_em_ = ~425 nm in vivo), click beetle green luciferase (CBG, λ_em_ = ~540 nm in vivo) and firefly luciferase (FLuc, λ_em_ = ~600 nm in vivo), using dorsal skin window chambers placed on tumor-bearing animals using four custom emission filters: 492 short pass (<489 nm), 540/50 band pass (491–591 nm), 594 long pass (>594 nm), and 635 nm long pass (>634 nm) (Figure 1a). A spectrum was measured using the series of acquired emission filter images where a mean pure spectrum for each reporter was then generated (Figure 1b,c and Figure S1a–e). Next, we validated that our user-generated pure spectral library correction algorithm could unmix different luciferases that used the same substrate using window chamber tumor-bearing animals injected with two separate B16F10 melanoma tumor cell lines that stably expressed different bioluminescent reporters: one B16F10 cell line stably expressed a ubiquitin promoter-driven CBG reporter and the other B16F10 tumor cell line stably expressed a CMV promoter-driven FLuc reporter. Following a single D-luciferin injection, bioluminescence imaging was performed using the different emission filters. Regardless of the filters used, CBG was unable to be fully spectrally separated from FLuc with the filters alone (Figure 1d). Only when using our user-generated pure spectral library corrections were the CBG-expressing tumors completely spectrally unmixed from the FLuc-expressing tumors (Figure 1e). The contrast-to-noise ratio (CNR) of the unmixed image in Figure 1e for CBG was 18.8 and for FLuc was 12.4, compared to the CNRs of 16.5, 1.7, and 3.6, respectively, for the raw emission filter images from the 491–591 nm bandpass filter, the >634 nm filter, and the open (no filter) images, demonstrating the enhanced signal separation afforded by spectral unmixing using user-generated pure spectrum corrections.

### 3.2. Imaging Reporters with Different Substrates

**Figure 2:**
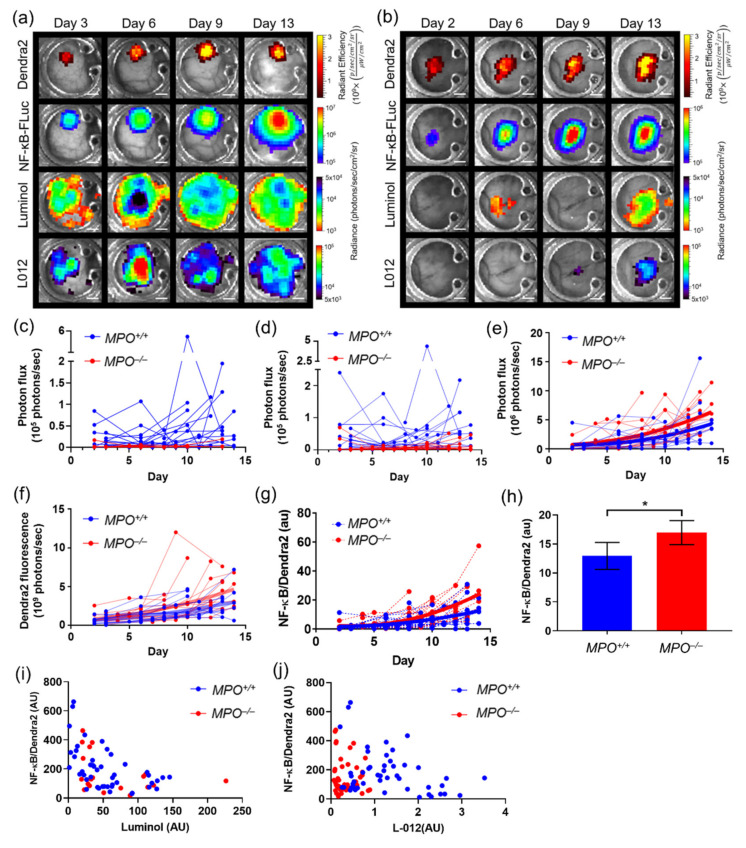
In vivo bioluminescence unmixing and quantification of different reporters in window chamber-bearing wild type (*MPO^+/+^*) and syngeneic MPO-null (*MPO^−/−^*) animals. Representative unmixed in vivo window chamber macro-imaging over time of B16F10 NF-κB-FLuc Dendra2 tumor growth in (**a**) *MPO^+/+^* and (**b**) *MPO^−/−^* animals (*n* = 17 *MPO^+/+^* animals, *n* = 16 *MPO^−/−^* animals). B16F10 tumor cells imaged using Dendra2 fluorescence (λ_ex_ = 409 nm, λ_em_ = 507 nm) and tumor NF-κB transcriptional activation monitored by FLuc bioluminescence following D-luciferin injection. Innate immune cell infiltration imaged using luminol and L-012; scale bar represents 2 mm. Window chamber longitudinal bioluminescence quantification as a function of time of (**c**) luminol and (**d**) L-012 in *MPO^+/+^* (blue) and *MPO^−/−^* (red) animals (each line represents a different animal). Quantification of (**e**) tumor NF-κB transcriptional activation as a function of time in individual animals (second-order polynomial non-linear regression with significantly different curve fits for *MPO^+/+^* versus *MPO^−/−^* data sets, *P* < 0.05, modified from previously reported publication [42]), (**f**) tumor Dendra2 fluorescence over time in each individual animal (second-order polynomial non-linear regression with significantly different curve fits for *MPO^+/+^* versus *MPO^−/−^* data sets, *p* < 0.05, modified from previously reported publication [42]), and (**g**) NF-κB transcriptional activation normalized to tumor mass as a function of time in individual animals (second-order polynomial non-linear regression with significantly different curve fits for *MPO^+/+^* versus *MPO^−/−^* data sets, *p* < 0.0006, modified from previously reported publication [42]). (**h**) Time-averaged mean of NF-κB transcriptional activation normalized to tumor mass using Dendra2 fluorescence in *MPO^+/+^* vs. *MPO^−/−^* animals (statistical significance calculated by identifying outliers, unpaired two-tailed Mann–Whitney *t* test, * *p* < 0.05, modified from previously reported publication [42]). Correlation of NF-κB tumor transcriptional activation and (**i**) luminol and (**j**) L-012 bioluminescence of *MPO^+/+^* (blue) and *MPO^−/−^* (red) animals (modified from previously reported publication [42]).

Because tumors grow heterogeneously, it is important to normalize bioluminescence signaling dynamics to tumor mass. One method to monitor tumor mass is using a dual fluorescent–bioluminescent reporter system. Here, B16F10 melanoma cells were generated to stably express a CMV-driven Dendra2 fluorophore (Dendra2; λ_ex_ = 409 nm, λ_em_ = 507 nm) as well as an NF-κB promoter-driven FLuc reporter (NF-κB-FLuc), where the FLuc bioluminescence magnitude and duration corresponded to the level of NF-κB transcriptional activation (B16F10 NF-κB-FLuc Dendra2). Using skin window chamber animals inoculated with B16F10 NF-κB-FLuc Dendra2 cells, bioluminescence and fluorescence imaging within the same imaging session sequentially at multiple time points in the same individual was demonstrated (Figure 2a,b). It should be noted that any desired fluorescence imaging should occur prior to bioluminescence imaging as there are known fluorescent properties of luciferins and redox reporters [38] which could complicate interpreting fluorescence signals. In addition, the photons released by circulating D-luciferin interacting with luciferases, although low, could all be detected by fluorescence mode imaging for several hours post injection. Immune cell infiltrates were monitored using two exogenous bioluminescent reporters, luminol and L-012. The oxidation of luminol by myeloperoxidase (MPO) activity releases blue photons, enabling the imaging of enzymatically active MPO produced from innate immune cells [39], whereas the oxidation of L-012, an analog of luminol, is induced by reactive oxygen species, reactive nitrogen species, and MPO, and also releases blue photons [40]. Luminol and L-012 can be resolved biochemically from each other using imaging sessions separated in time by at least 20 min. The time dynamics of luminol and L-012 were similar and cleared from animals within 30 min, as had been previously reported (Figure S1f) [39,41]. Thus, luminol/L-012 and luciferases can be further spectrally unmixed both biochemically and by using a user-generated spectral unmixing library. 

The power of the intravital bioluminescence platform is demonstrated by comparing tumor cell NF-κB transcriptional activation and immune cell tracking during cancer progression. Wild type (*MPO^+/+^*) and syngeneic MPO-null (*MPO^−/−^*) animals with skin window chambers were injected subcutaneously with B16F10 NF-κB-FLuc Dendra2 reporter cells and optically imaged over time (Figure 2a,b). Spatial and temporal resolutions of immune infiltrate, tumor growth, and tumor NF-κB transcriptional activation were monitored during tumor progression. As expected, Dendra2 fluorescence increased over time, indicative of an increase in tumor size. NF-κB-driven bioluminescence also increased as the tumor increased. Immune infiltration was variable and changed both spatially and temporally during tumor progression regardless of host animal phenotype (Figure 2a–d). As expected, luminol and L-012 bioluminescence signals were significantly decreased in the *MPO^−/−^* animals (Figure 2c,d; reformatted and reanalyzed from a previously reported publication [42]). Endpoint immunohistology of window chamber skin flaps confirmed NF-κB protein levels and myeloid cell infiltration using CD11b and Ly6G staining (Figure S2a).

To quantify the spatial dynamics from macro-imaging over time, window chamber images required rotational reorientation and registration to a fixed reference position. We developed a macro in ImageJ that used the bolts from the window chambers to orient the windows to a fixed reference coordinate (Figure S2b, ImageJ code provided in supplementary materials). The image was then cropped to the size of the glass cover slip of the window chamber (Figure S2c). This was done for each image with each reporter, resulting in cropped and aligned images for all reporters at every time point (Figure S2d). Post-processing of images using our ImageJ macro allowed not only temporal but also spatial distribution of imaging signals to be quantified using different reporters. For example, the overlap and differences between luminol and L-012 photon flux could be quantified by pointwise subtraction (Figure S2e) or division (Figure S2f) of L-012 from luminol. Similar images resulted regardless of analysis by luminol subtraction or division, wherein the enhanced photon flux (>0 in subtraction image Figure S2e, or >1 in the division image Figure S2f) was likely due to the oxidation of L-012 by additional reactive oxygen species or reactive nitrogen species within the environment. 

Quantifying the NF-κB transcriptional activation over time showed an increase in NF-κB activation in *MPO^−/−^* mice compared to wild type *MPO^+/+^* mice (Figure 2e; reformatted and reanalyzed from our previously reported publication [42]. However, quantifying the Dendra2 fluorescence, indicative of tumor growth over time, also demonstrated an increase in tumor growth in *MPO^−/−^* animals (Figure 2f; reformatted and reanalyzed from our previously reported publication [42]). To ensure that the increase in NF-κB activation was not merely due to increased tumor growth in *MPO^−/−^* animals, NF-κB transcriptional activation was normalized to tumor mass using Dendra2 fluorescence imaging. Due to the heterogeneity of tumor growth, spatial distribution of signal was also required to be considered; thus, following processing by the ImageJ macro to orient and crop all images, the NF-κB-FLuc BLI image was divided pixel by pixel by the corresponding Dendra2 fluorescence image (Figure S2g). Thus, the inclusion of a constitutively active reporter, such as Dendra2, provided a method to normalize signaling dynamics to tumor mass. Normalized NF-κB activation per unit tumor mass as a function of time and stepwise time average demonstrated an increase in specific NF-κB transcriptional activation within B16F10 tumors grown in *MPO^−/−^* animals compared to *MPO^+/+^* animals (Figure 2g,h; reformatted and reanalyzed from a previously reported publication [42]). Interestingly, the normalized NF-κB-FLuc per unit tumor mass demonstrated heterogeneous spatial activation of NF-κB within the tumor during development (Figure S2g). More complicated algorithms, in principle, could be applied to the aligned, cropped images to further quantify spatial and temporal features of the images. For example, using a Gaussian mixture model initialized with a k-means clustering algorithm and regularized by a Markov random field calculated the Pearson correlation coefficient of image signal intensity between NF-κB activation and luminol or L-012 in *MPO^+/+^* and *MPO^−/−^* animals (Figure 2i,j; reformatted and reanalyzed from a previously reported publication [42]). A negative correlation was found between both luminol and L-012 with NF-κB activation, but only in *MPO^+/+^* animals (Pearson coefficients of r = −0.53 (*p* < 0.0001) and r = −0.32 (*p* < 0.05)), while no significant correlation was found in *MPO^−/−^* animals [42]. The biological mechanism of this correlation has been fully discussed previously [42].

**Figure 3:**
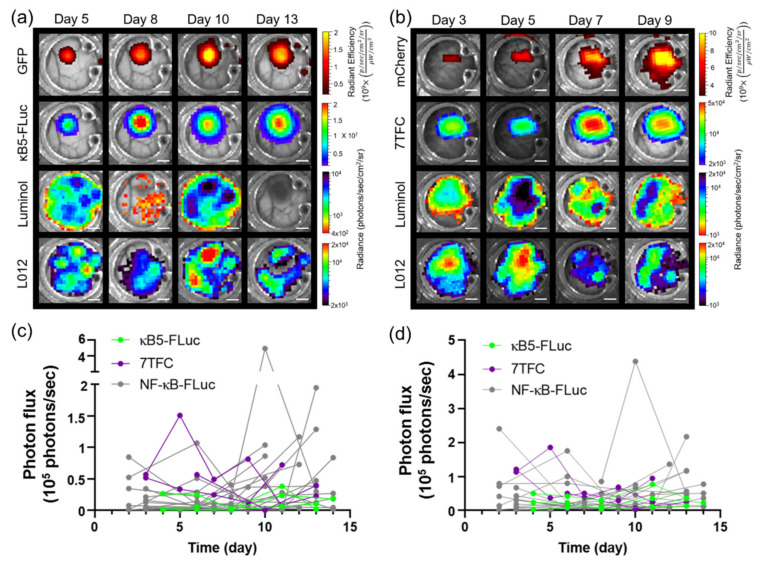
In vivo bioluminescence unmixing in wild type window chamber-bearing animals with different B16F10 signaling reporter cells. Representative unmixed in vivo window chamber imaging over time of (**a**) B16F10 κB5-FLuc GFP reporter cells (*n* = 3 animals) and (**b**) B16F10 7TFC-mCherry reporter cells (*n* = 4 animals). GFP or mCherry fluorescence monitored B16F10 tumor growth. Reporter activation was monitored by FLuc bioluminescence (κB5-Fluc or 7TFC) following D-luciferin i.p. injection. Innate immune cell infiltration imaged by bioluminescence using luminol and L-012; scale bar represents 2 mm. Longitudinal bioluminescence quantification as a function of time of (**c**) luminol and (**d**) L-012 in window chamber-bearing animals with κB5-FLuc and 7TFC reporter cells (luminol and L-012 data for NF-κB-FLuc reporter cells included as reference).

The intravital bioluminescence platform can be used with a variety of bioluminescent and fluorescent signaling reporters. For example, B16F10 melanoma cells stably expressing a concatenated κB5 promoter-driven FLuc reporter (κB5-FLuc) that also monitors NF-κB activation and a constitutively active IRES-driven green fluorescent protein (GFP) were resolved from each other as well as luminol and L-012 imaging over time during tumor progression (Figure 3a). Additionally, B16F10 tumors stably expressing a 7TFC reporter that monitors Wnt-β-cat activation [34] through FLuc bioluminescence and a constitutively active mCherry fluorophore driven by SV40 were spectrally unmixed from luminol and L-012 imaging monitored over time during tumor progression (Figure 3b). Immune infiltration was variable and changed both spatially and temporally during tumor progression regardless of the reporter expressed by B16F10 cells (Figure 3c,d).

### 3.3. Limitations of Spectral Unmixing Luciferase–Luciferin Reporters In Vivo

There are limitations in the spectral unmixing capabilities of our intravital macro-imaging platform. B16F10 melanoma cells were generated to stably express dual luciferase–luciferin reporters, the NF-κB promoter-driven FLuc reporter (NF-κB-FLuc) and a constitutively active ubiquitin promoter-driven CBG reporter, both described above (B16F10 NF-κB-FLuc CBG). When using bioluminescent reporters that use the same substrate, if one reporter generates photons an order of magnitude greater than the other reporter, there are challenges during spectral unmixing. In the B16F10 NF-κB-FLuc CBG tumors, the NF-κB-FLuc reporter photonic output was an order of magnitude lower than the constitutively active CBG reporter, particularly at the early time points (Figure S3). This difference in the photon flux of the reporters limited the acquisition time of the reporter with the lower photon production, in this case NF-κB-FLuc, resulting in decreased sensitivity of detecting NF-κB activation. Furthermore, in macro-imaging, photon detection was cumulative, resulting in the center of the tumor (which has the greatest tumor mass) having the highest photon production. Because of the difference in magnitude of the photon production from the two reporters, CBG photon production dominated and, thus, minimal FLuc photons were observed (Figure S3). In this example, when normalizing the transcriptional activation of NF-κB to the metabolism of the cell based upon CBG photon flux, a gradient frequently appeared from the center of the tumor radially, which might have indicated that cells at the tumor periphery had higher NF-κB activation (Figure S3 NF-κB-FLuc/CBG). However, this was most likely a technical artifact, the result of minimal FLuc photons being detected due to the large differences in photon production of the two bioluminescent reporters, particularly at the early tumor time points. When we compare the NF-κB activation per tumor mass in B16F10 NF-κB-FLuc Dendra2 tumors, a heterogeneous spatial distribution of NF-κB activation was observed and only at later tumor time points was NF-κB activation observed at the tumor periphery (Figure S2g). Complicating matters further, deep tissue CBG photons can appear red-shifted at the surface due to enhanced photonic absorption of green photons at depth, which would result in relatively more red photons being detected in the FLuc red channel; thus, the detectable photons in the NF-κB-FLuc channel could actually be red-shifted CBG photons bleeding through, competing with the low output of photons by FLuc in this case, for which the unmixing algorithm performs poorly (Figure S3). Therefore, it is important when using a dual luciferase–luciferin reporter system to ensure paired photon productions are approximately matched or of the same relative magnitude. 

### 3.4. Imaging Using Transgenic Animals

**Figure 4:**
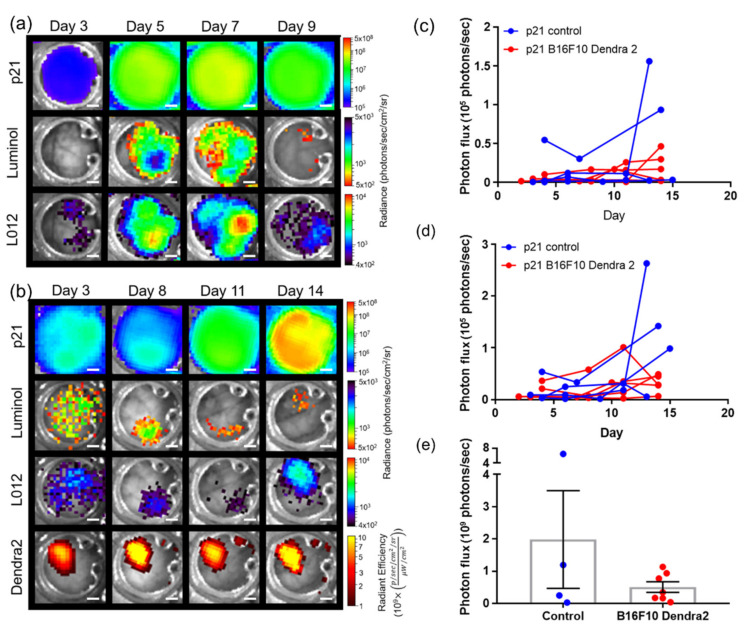
In vivo bioluminescence unmixing in *p21-FLuc* transgenic skin window chamber animals (**a**) without (*n* = 4 animal) and (**b**) with B16F10 Dendra2 reporter cells (*n* = 6 animals). Dendra2 fluorescence monitors B16F10 tumor growth. Reporter p21 activation is monitored by FLuc bioluminescence following D-luciferin i.p. injection. Innate immune cell infiltration imaged by bioluminescence using luminol and L-012; scale bar represents 2 mm. Window chamber bioluminescence quantification of (**c**) luminol, (**d**) L-012, and (**e**) p21 activation in *p21-FLuc* window chamber-bearing animals without (blue) and with B16F10 Dendra2 reporter cells (red).

Spectral unmixing can also resolve signals in transgenic reporter mice. As an example, skin window chambers were implanted on knock-in p21 luciferase (*p21-FLuc*) reporter mice [35] with and without B16F10 tumor cells stably expressing the CMV-driven Dendra2 fluorophore. Bioluminescence imaging in *p21-FLuc* animals monitored endogenous p21 promoter activity [35]. p21 promoter-driven FLuc in the host was spectrally resolved from interstitial luminol and L-012 imaging, demonstrating spatial and temporal resolution of p21 activation and immune infiltrate (Figure 4a,b). Quantification of luminol and L-012 imaging in animals without and with B16F10 tumors demonstrated no significant difference in the dynamics of the immune infiltrate temporally (Figure 4c,d). However, surprisingly, p21 activity was reduced in tumor-bearing animals compared to healthy animals (Figure 4e).

### 3.5. Imaging with Different Window Chambers

**Figure 5:**
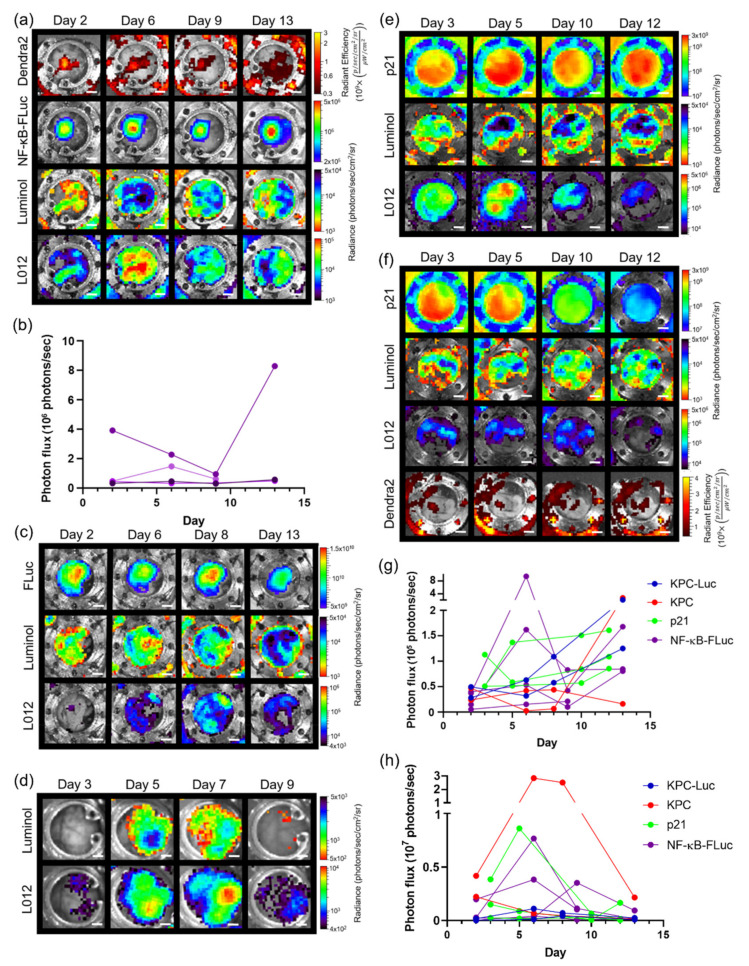
In vivo bioluminescence unmixing in wild type animals with a pancreas window chamber. (**a**) Representative unmixed in vivo pancreas window chamber macro-imaging over time of Pan02 tumor growth; Pan02 tumor cells imaged using Dendra2 fluorescence (λ_ex_ = 409 nm, λ_em_ = 507 nm) and tumor NF-κB transcriptional activation monitored by FLuc bioluminescence following D-luciferin i.p. injection. (**b**) Corresponding longitudinal bioluminescence quantification as a function of time of NF-κB transcriptional activation in pancreas window chamber animals with Pan02 NF-κB-FLuc Dendra2 reporter cells (*n* = 4 animals); each colored line corresponds to the NF-κB bioluminescent reporter output in an individual animal. In vivo bioluminescence unmixing in spontaneous pancreas ductal adenocarcinoma animal model. Representative unmixed bioluminescence images of abdominal window chambers (implanted at week 14) visualizing the pancreas in (**c**) *KPC-Luc* and (**d**) *KPC* control animals. Pancreas tumor growth monitored by FLuc bioluminescence following D-luciferin i.p. injection. In vivo bioluminescence unmixing in transgenic *p21-FLuc* pancreas window chamber animals (**e**) without (*n* = 2 animals) and (**f**) with Pan02 Dendra2 reporter cells (*n* = 2 animals). Reporter p21 activation is monitored by FLuc bioluminescence following D-luciferin injection. Innate immune cell infiltration imaged by bioluminescence using luminol and L-012. Longitudinal bioluminescence quantification as a function of time of (**g**) luminol and (**h**) L-012 of pancreas window chamber animals. Scale bar represents 2 mm.

This multi-modal, multi-spectral imaging platform was not limited to skin window chambers. For example, using an abdominal window chamber, the pancreas and orthotopic pancreatic tumor growth of Pan02 cancer cells stably expressing both the NF-κB transcriptional activation reporter and the Dendra2 fluorophore (Pan02 NF-κB-FLuc Dendra2) were imaged. Tumor growth and NF-κB activation were resolved from immune infiltrate imaging (Figure 5a). Again, luminol and L-012 signaling spatially and temporally changed during tumor progression. Bioluminescence quantification of NF-κB transcriptional activation was also measured as a function of time (Figure 5b). Furthermore, the spontaneous pancreas ductal adenocarcinoma animal (PDAC) model, *LSL-Kras^G12D/+^; LSL-p53^T172H/+^; Pdx1-Cre* (KPC), was bred with *ROSA26-pGAGGs-LSL* luciferase animals, resulting in a spontaneous bioluminescent PDAC animal model (*KPC-Luc*) in which spontaneous pancreas tumors developed that were bioluminescent [36]. *KPC-Luc* animals develop pancreatic tumors around 100–140 days of age [36]. Abdominal window chambers were implanted on *KPC-Luc* and *KPC* control (no bioluminescent reporter) animals at week 14. Herein, pancreas tumor growth was monitored by FLuc and spectrally resolved from luminol and L-012 imaging (Figure 5c). Regardless of the presence of tumors, immune infiltration changed spatially and temporally (Figure 5c,d). Using abdominal window chambers for imaging, the pancreas was also imaged using *p21-FLuc* transgenic animals without and with Pan02 cancer cells stably expressing a Dendra2 fluorophore (Figure 5e,f). Similar to the skin window chamber models, bioluminescence could be quantified; luminol and L-012 imaging was variable regardless of using tumor or transgenic models (Figure 5g,h). Luminol photon flux was in the same order of magnitude regardless of window chamber location (Figure S4a). However, the photon flux detected in the pancreas window chamber was over ten-fold higher using L-012 compared to the skin window chamber, suggesting that a larger influx of immune infiltrates and/or higher reactive oxygen and nitrogen species were present in the abdomen (gut) compared to the skin (Figure S4b) [43,44]. Bioluminescence quantification within the pancreas window chamber animals also demonstrated a decrease in p21 activity in the presence of Pan02 tumors compared to control animals (Figure S4c,d).

## 4. Discussion

This study demonstrated the potential application of in vivo spectral unmixing using multiple bioluminescent reporters. Furthermore, bioluminescence imaging could be combined with fluorescence imaging, which could have complementary roles, thereby providing a highly sensitive in vivo whole-body molecular imaging platform. As we have demonstrated, this platform could be used to image in real time the interaction between cancer cell signaling dynamics and immune cell enzymatic activities during tumor progression and could be further extended to other diseases in vivo. With the continued development of genetically encoded imaging reporters that monitor changes in transcription, translation, protein folding, protein association, and protein degradation as well as second messengers, these examples illustrate the signaling networks and molecular/cellular processes that might be studied and are only limited by the number of imaging channels. Herein, we demonstrated that this methodology could resolve four imaging reporters (three bioluminescent reporters and one fluorescent reporter) through a combination of multi-spectral and multi-modal acquisition and analysis in real time. In order to achieve bioluminescence spectral unmixing, the inclusion of custom emission filters in our small animal bioluminescence imaging system was necessary. With the continued development of a broader array of different emissions for bioluminescent reporters, and the combination of bioluminescence and fluorescence, we anticipate more channels will be readily imagable. For example, spectral unmixing of bioluminescence could be increased to four imaging reporters with the inclusion of far-red click beetle red luciferase mutants ((λ_em_ > 650 nm) [45], which can be spectrally resolved from luminol ((λ_em_ = ~425 nm), CBG ((λ_em_ = ~540 nm), and FLuc ((λ_em_ = ~600 nm). This technique will likely be extensible to more reporters as the spectral unmixing methodology could be further applied to fluorescent reporters. This intravital molecular imaging platform, which combines different emission bioluminescent and fluorescent genetically encoded and exogenous reporters, provides a powerful strategy to study molecular and cellular processes in real time in intact living systems. The use of our molecular macro-imaging platform provides a strategy to begin to understand how cells communicate both spatially and temporally in a quantitative manner in a heterogeneous living system in vivo. The sensitivity of the macro-imaging strategy provides complimentary information to our microscopic platform [14]; the same window chamber implant can be imaged at both the macro- and microscopic scale, consecutively monitoring low photon producing reporters globally as well as molecular and cellular changes at high resolution. We demonstrate the capacity of this intravital molecular imaging platform to image tumor signaling dynamics concurrently with immune cell enzyme activities while monitoring tumor growth in real time. Tumor–immune interactions are now accepted as an important factor relevant for the clinical management of patients with cancer [1,2,3,4,5]. Strategies to understand these interaction networks between tumor cells and immune cells are therefore critical to ensure successful advances in disease intervention and response.

## Data Availability

All data presented within this study are available within the manuscript and the Supplementary Materials.

## Supplementary Materials

The following are available online at https://www.mdpi.com/2073-4409/10/3/489/s1: Figure S1. Generation of pure bioluminescent spectra in vivo for spectral unmixing. Figure S2. Representative immunohistology (a) of MPO+/+ and MPO-/- window chamber animal skin flaps at endpoint showing H&E, NF-κB, CD11b and Ly6G staining confirming NF-κB protein levels and presence of myeloid cells (scale bar, 4 mm). Figure S3. Challenges in BLI spectral unmixing (n = 3 animals) in window chamber animals using B16F10 NF-κB-FLuc CBG tumors. Figure S4. Bioluminescence quantification of (a) luminol and (b) L-012 signal comparing skin (n = 33, purple) and pancreas (n = 11, green) window chambers. Pancreas window chamber bioluminescent quantification of (c) p21 as a function of time, (d) time average of mean p21 activation in p21-FLuc pancreas window chamber-bearing animals without (blue) and with Pan02 Dendra2 reporter cells (red); n = 2 animals.

## References

[1] Binnewies M., Roberts E.W., Kersten K., Chan V., Fearon D.F., Merad M., Coussens L.M., Gabrilovich D.I., Ostrand-Rosenberg S., Hedrick C.C. (2018). Understanding the tumor immune microenvironment (TIME) for effective therapy. Nat. Med..

[2] Smyth M.J., Ngiow S.F., Ribas A., Teng M.W.L. (2016). Combination cancer immunotherapies tailored to the tumour microenvironment. Nat. Rev. Clin. Oncol..

[3] Hanahan D., Weinberg R.A. (2011). Hallmarks of Cancer: The Next Generation. Cell.

[4] Schreiber R.D., Old L.J., Smyth M.J. (2011). Cancer Immunoediting: Integrating Immunity’s Roles in Cancer Suppression and Promotion. Science.

[5] Quail D.F., Joyce J. (2013). Microenvironmental regulation of tumor progression and metastasis. Nat. Med..

[6] Gross S., Piwnicaworms D. (2005). Spying on cancer: Molecular imaging in vivo with genetically encoded reporters. Cancer Cell.

[7] Prescher J., Contag C.H. (2010). Guided by the light: Visualizing biomolecular processes in living animals with bioluminescence. Curr. Opin. Chem. Biol..

[8] Mezzanotte L., van’t Root M., Karatas H., Goun E.A., Löwik C.W. (2017). In Vivo Molecular Bioluminescence Imaging: New Tools and Applications. Trends Biotechnol..

[9] Kocher B., Piwnica-Worms D. (2013). Illuminating Cancer Systems with Genetically Engineered Mouse Models and Coupled Luciferase Reporters In Vivo. Cancer Discov..

[10] Stacer A.C., Nyati S., Moudgil P., Iyengar R., Luker K.E., Rehemtulla A., Luker G.D. (2013). NanoLuc reporter for dual luciferase imaging in living animals. Mol. Imaging.

[11] Daniel C., Poiret S., Dennin V., Boutillier D., Lacorre D.A., Foligné B., Pot B. (2015). Dual-Color Bioluminescence Imaging for Simultaneous Monitoring of the Intestinal Persistence of Lactobacillus plantarum and Lactococcus lactis in Living Mice. Appl. Environ. Microbiol..

[12] Mezzanotte L., Que I., Kaijzel E., Branchini B., Roda A., Löwik C. (2011). Sensitive Dual Color In Vivo Bioluminescence Imaging Using a New Red Codon Optimized Firefly Luciferase and a Green Click Beetle Luciferase. PLoS ONE.

[13] Gammon S.T., Leevy W.M., Gross S., Gokel G.W., Piwnica-Worms D. (2006). Spectral Unmixing of Multicolored Bioluminescence Emitted from Heterogeneous Biological Sources. Anal. Chem..

[14] Liu T.W., Gammon S.T., Piwnica-Worms D. (2021). Multi-modal multi-spectral intravital microscopic imaging of signaling dynamics in real-time during tumor-immune interactions. Cells.

[15] Gammon S.T., Liu T.W., Piwnica-Worms D. (2020). Interrogating Cellular Communication in Cancer with Genetically Encoded Imaging Reporters. Radiol. Imaging Cancer.

[16] Pittet M.J., Weissleder R. (2011). Intravital Imaging. Cell.

[17] Palmer G.M., Fontanella A.N., Shan S., Hanna G., Zhang G., Fraser C.L., Dewhirst M.W. (2011). In vivo optical molecular imaging and analysis in mice using dorsal window chamber models applied to hypoxia, vasculature and fluorescent reporters. Nat. Protoc..

[18] Kircher M.F., Gambhir S.S., Grimm J. (2011). Noninvasive cell-tracking methods. Nat. Rev. Clin. Oncol..

[19] Entenberg D., Voiculescu S., Guo P., Borriello L., Wang Y., Karagiannis G.S., Jones J., Baccay F., Oktay M., Condeelis J. (2018). A permanent window for the murine lung enables high-resolution imaging of cancer metastasis. Nat. Methods.

[20] Harney A.S., Arwert E.N., Entenberg D., Wang Y., Guo P., Qian B.-Z., Oktay M.H., Pollard J.W., Jones J.G., Condeelis J.S. (2015). Real-Time Imaging Reveals Local, Transient Vascular Permeability, and Tumor Cell Intravasation Stimulated by TIE2hi Macrophage–Derived VEGFA. Cancer Discov..

[21] Rodell C.B., Arlauckas S.P., Cuccarese M.F., Garris C.S., Li R., Ahmed M.S., Kohler R.H., Pittet M.J., Weissleder R. (2018). TLR7/8-agonist-loaded nanoparticles promote the polarization of tumour-associated macrophages to enhance cancer immunotherapy. Nat. Biomed. Eng..

[22] Suijkerbuijk S.J., Van Rheenen J. (2017). From good to bad: Intravital imaging of the hijack of physiological processes by cancer cells. Dev. Biol..

[23] Lohela M., Casbon A.-J., Olow A., Bonham L., Branstetter D., Weng N., Smith J., Werb Z. (2014). Intravital imaging reveals distinct responses of depleting dynamic tumor-associated macrophage and dendritic cell subpopulations. Proc. Natl. Acad. Sci. USA.

[24] Lohela M., Werb Z. (2010). Intravital imaging of stromal cell dynamics in tumors. Curr. Opin. Genet. Dev..

[25] Park J., Wysocki R.W., Amoozgar Z., Maiorino L., Fein M.R., Jorns J., Schott A.F., Kinugasa-Katayama Y., Lee Y., Won N.H. (2016). Cancer cells induce metastasis-supporting neutrophil extracellular DNA traps. Sci. Transl. Med..

[26] Fein M.R., Egeblad M. (2013). Caught in the act: Revealing the metastatic process by live imaging. Dis. Model. Mech..

[27] Figley S.A., Chen Y., Maeda A., Conroy L., McMullen J.D., Silver J.I., Stapleton S., Vitkin A., Lindsay P., Burrell K. (2013). A Spinal Cord Window Chamber Model for In Vivo Longitudinal Multimodal Optical and Acoustic Imaging in a Murine Model. PLoS ONE.

[28] Rouffiac V., Roux K.S., Salomé-Desnoulez S., Leguerney I., Ginefri J., Sébrié C., Jourdain L., Lécluse Y., Laplace-Builhé C. (2020). Multimodal imaging for tumour characterization from micro- to macroscopic level using a newly developed dorsal chamber designed for long-term follow-up. J. Biophotonics.

[29] Souris J.S., Hickson J., Msezane L., Rinker-Schaeffer C.W., Chen C.-T. (2013). Flexible peritoneal windows for quantitative fluorescence and bioluminescence preclinical imaging. Mol. Imaging.

[30] Moss B.L., Gross S., Gammon S.T., Vinjamoori A., Piwnica-Worms D. (2008). Identification of a ligand-induced transient refractory period in nuclear factor-kappaB signaling. J. Biol. Chem..

[31] Moss B.L., Elhammali A., Fowlkes T., Gross S., Vinjamoori A., Contag C.H., Piwnica-Worms D. (2012). Interrogation of Inhibitor of Nuclear Factor κB α/Nuclear Factor κB (IκBα/NF-κB) Negative Feedback Loop Dynamics. J. Biol. Chem..

[32] Warrington N.M., Gianino S.M., Jackson E., Goldhoff P., Garbow J.R., Piwnica-Worms D., Gutmann D.H., Rubin J.B. (2010). Cyclic AMP Suppression Is Sufficient to Induce Gliomagenesis in a Mouse Model of Neurofibromatosis-1. Cancer Res..

[33] Gross S., Piwnica-Worms D. (2005). Real-time imaging of ligand-induced IKK activation in intact cells and in living mice. Nat. Chem. Biol..

[34] Fuerer C., Nusse R. (2010). Lentiviral Vectors to Probe and Manipulate the Wnt Signaling Pathway. PLoS ONE.

[35] Tinkum K.L., Marpegan L., White L.S., Sun J., Herzog E.D., Piwnica-Worms D. (2011). Bioluminescence Imaging Captures the Expression and Dynamics of Endogenous p21 Promoter Activity in Living Mice and Intact Cells. Mol. Cell. Biol..

[36] Zhao J., Wang H., Hsiao C.-H., Chow D.S.-L., Koay E.J., Kang Y., Wen X., Huang Q., Ma Y., Bankson J.A. (2018). Simultaneous inhibition of hedgehog signaling and tumor proliferation remodels stroma and enhances pancreatic cancer therapy. Biomaterials.

[37] Ritsma L., Steller E.J., Ellenbroek S.I.J., Kranenburg O., Rinkes I.H.M.B., Van Rheenen J. (2013). Surgical implantation of an abdominal imaging window for intravital microscopy. Nat. Protoc..

[38] Gandelman O., Brovko L., Ugarova N., Chikishev A., Shkurimov A. (1993). Oxyluciferin fluorescence is a model of native bioluminescence in the firefly luciferin—luciferase system. J. Photochem. Photobiol. B Biol..

[39] Gross S., Gammon S.T., Moss B.L., Rauch D., Harding J.J., Heinecke J.W., Ratner L., Piwnica-Worms D. (2009). Bioluminescence imaging of myeloperoxidase activity in vivo. Nat. Med..

[40] Goiffon R.J., Martinez S.C., Piwnica-Worms D. (2015). A rapid bioluminescence assay for measuring myeloperoxidase activity in human plasma. Nat. Commun..

[41] Kielland A., Blom T., Nandakumar K.S., Holmdahl R., Blomhoff R., Carlsen H. (2009). In vivo imaging of reactive oxygen and nitrogen species in inflammation using the luminescent probe L-012. Free Radic. Biol. Med..

[42] Liu T.W., Gammon S.T., Yang P., Fuentes D., Piwnica-Worms D. (2021). HOCl is a paracrine effector linking myeloid cells to NF-kB signaling in melanoma by trans-inhibition of IKK. Sci. Signal..

[43] Conroy E., Aviello G. (2019). Imaging Intestinal ROS in Homeostatic Conditions Using L-012. Breast Cancer.

[44] Emani R., Asghar M.N., Toivonen R., Laurén L., Soderstrom M., Toivola D.M., Van Tol E.A.F., Hänninen A. (2013). Casein hydrolysate diet controls intestinal T cell activation, free radical production and microbial colonisation in NOD mice. Diabetologia.

[45] Hall M.P., Woodroofe C.C., Wood M.G., Que I., Root M.V., Ridwan Y., Shi C., Kirkland T.A., Encell L.P., Wood K.V. (2018). Click beetle luciferase mutant and near infrared naphthyl-luciferins for improved bioluminescence imaging. Nat. Commun..

