# Supplementary material for: Multi-Modal Multi-Spectral Intravital Macroscopic Imaging of Signaling Dynamics in Real Time during Tumor–Immune Interactions
Source: Cells. 2021 Feb 25;10(3):489. doi: 10.3390/cells10030489 (PMC7996138; doi:10.3390/cells10030489)
Supplement: Supplementary file 1 [file cells-10-00489-s001.pdf]

cells MDPI

1. Supplemental Materials

1.1. Supplemental Figure

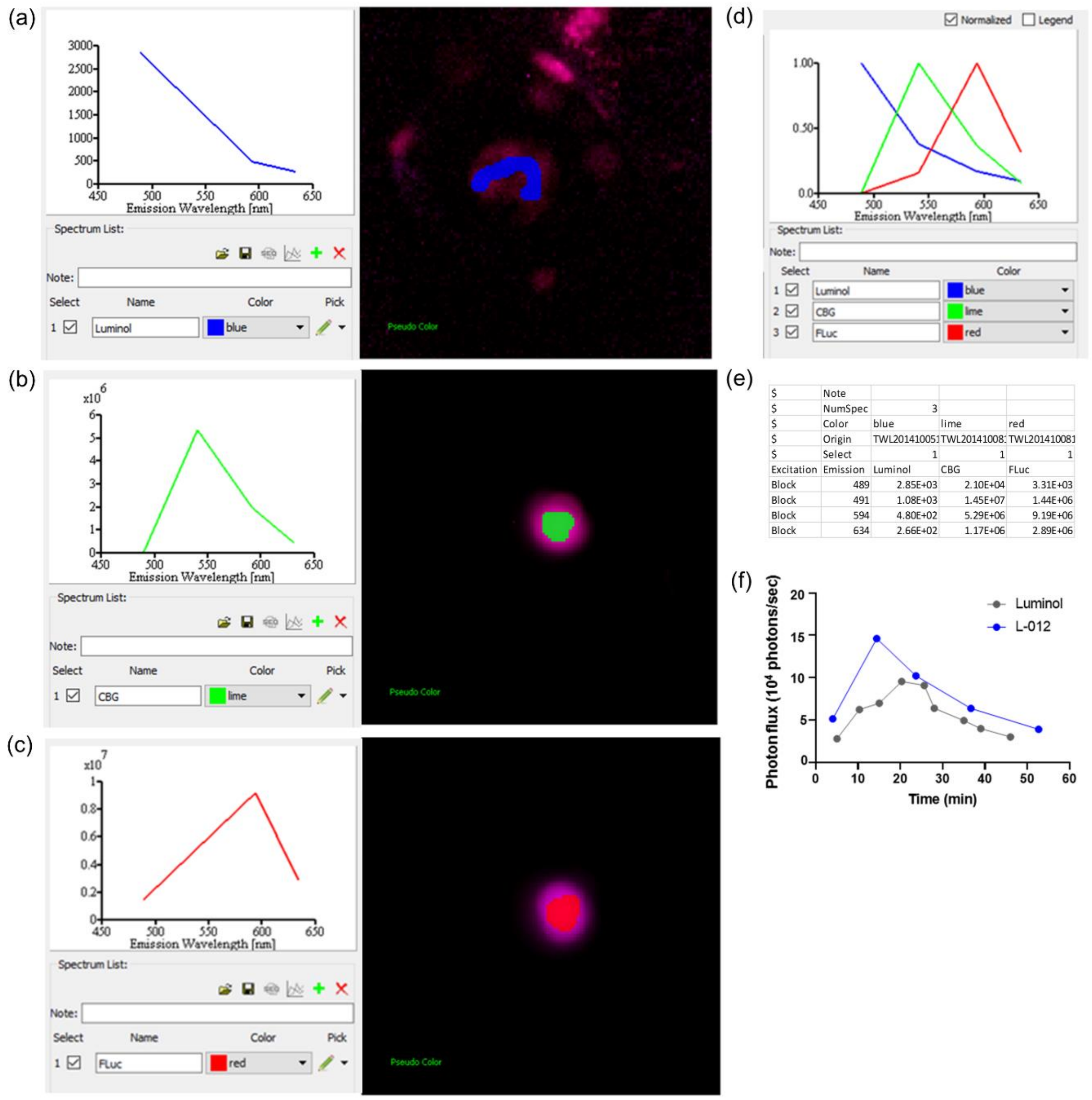

Figure S1. Generation of pure bioluminescent spectra *in vivo* for spectral unmixing. Representative raw emission spectra in vivo for (**a**) luminol, (**b**) click beetle green (CBG) and (**c**) firefly luciferase (FLuc). (**d**) User-generated spectral library to unmix luminol, CBG, and FLuc built from the average emission spectra in window chamber-bearing animals using luminol (n = 3 animals), CBG (n = 6 animals) or FLuc (n = 6 animals). (**e**) Raw data of the average emission spectra in user-generated spectral library for Living Image. (**f**) Dynamic temporal imaging of luminol and L-012 reporters in skin window chamber-bearing animals (n = 2 animals for each reporter).

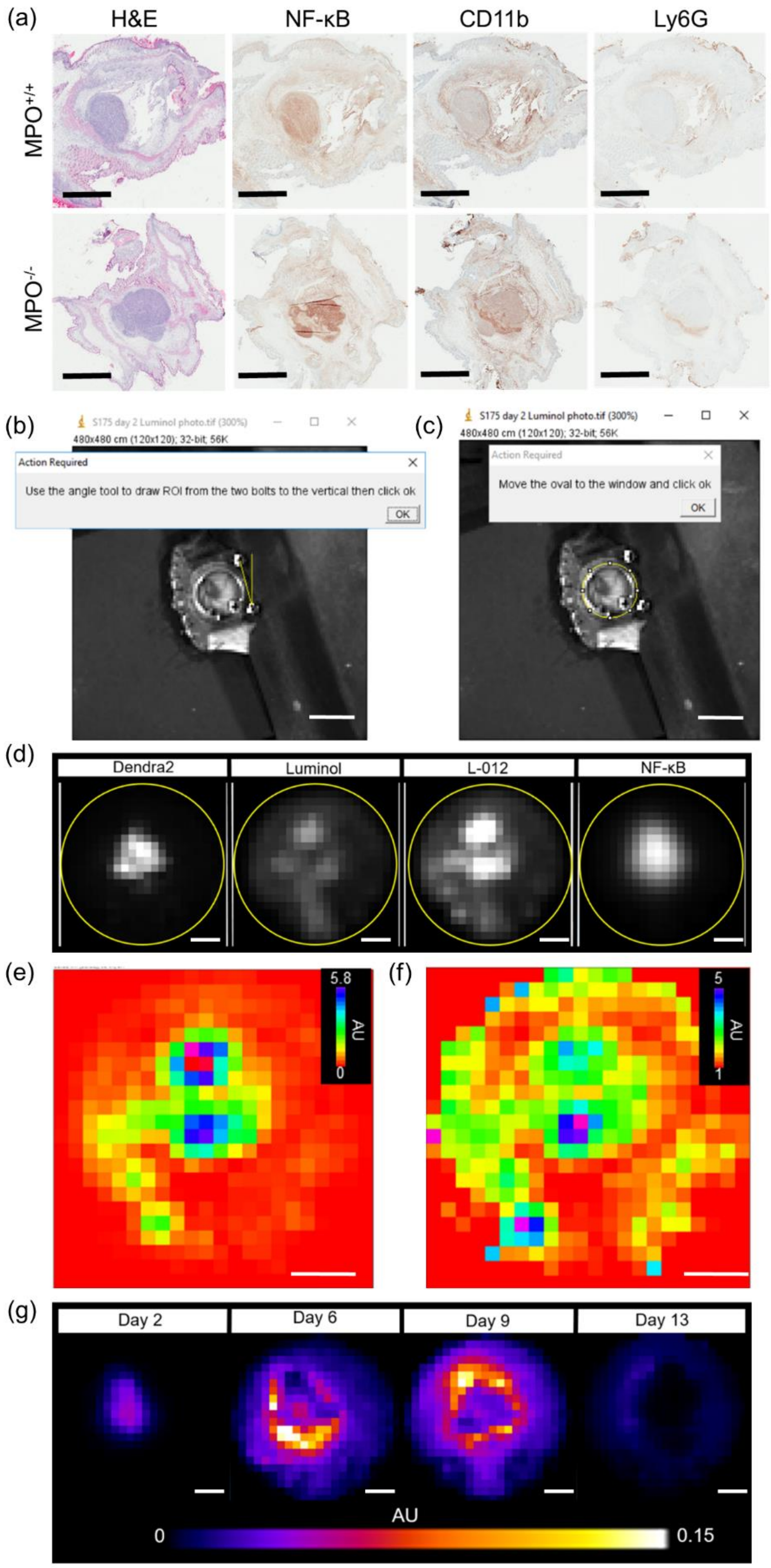

Figure S2. Representative immunohistology (**a**) of *MPO*$^{+/+}$ and *MPO*$^{-/-}$ window chamber skin flaps at endpoint showing H&E, NF-κB, CD11b and Ly6G staining confirming NF-κB protein levels and presence of myeloid cells (scale bar, 4 mm). (**b**,**c**) ImageJ macro to align window chamber for quantification. Representative image of ImageJ macro for (**b**) aligning and (**c**) cropping window chamber. (**d**) Representative image of a single time point of window chamber imaging post processing

with ImageJ macro. Representative images of the overlap and differences between luminol and L-012 images by (**e**) pixelwise subtraction or (**f**) division. (**g**) Representative image of the same animal over time of NF-κB-Fluc pixelwise division by Dendra2 fluorescence.

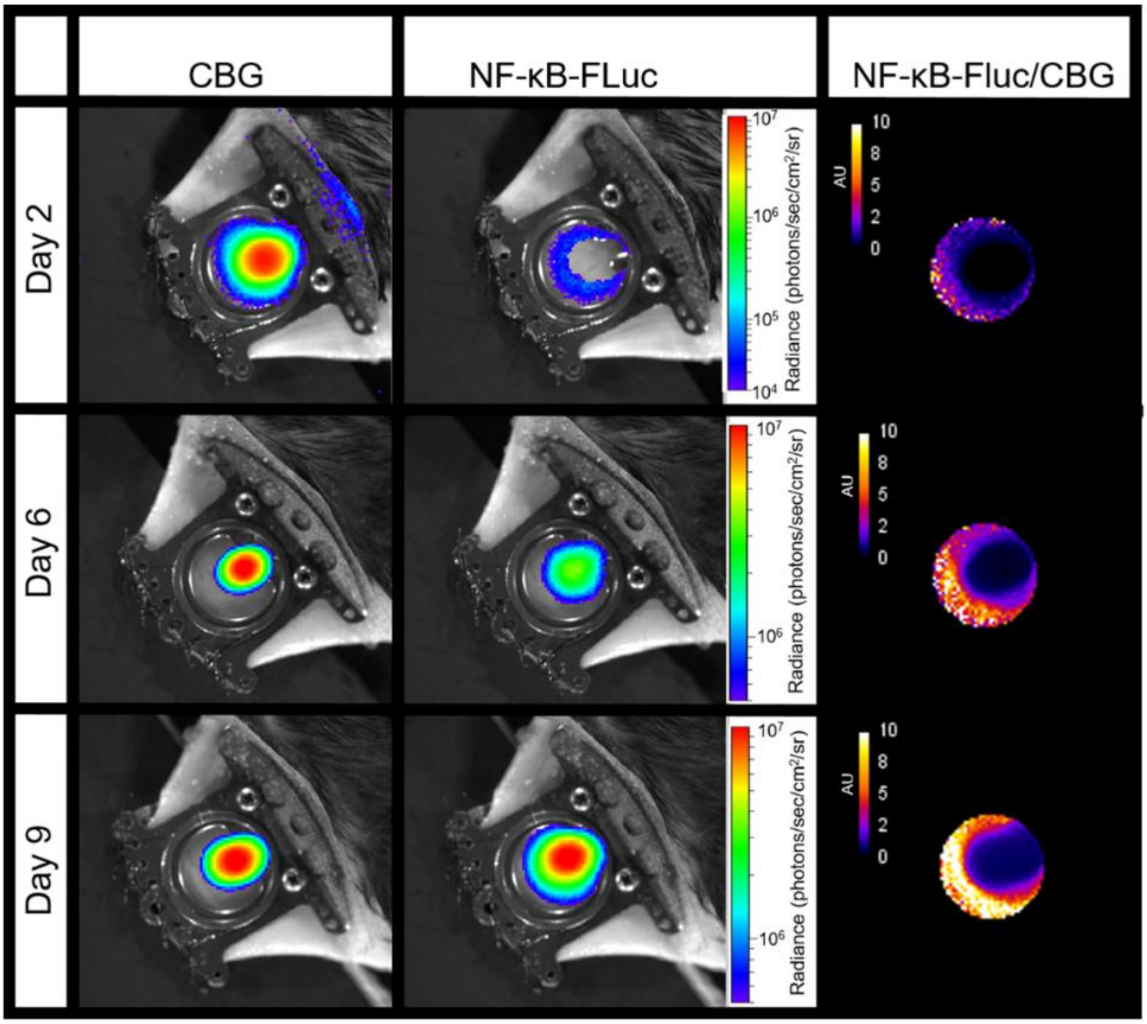

Figure S3. Challenges in BLI spectral unmixing (n = 3 animals) in window chamber animals using B16F10 NF-κB-FLuc CBG tumors.

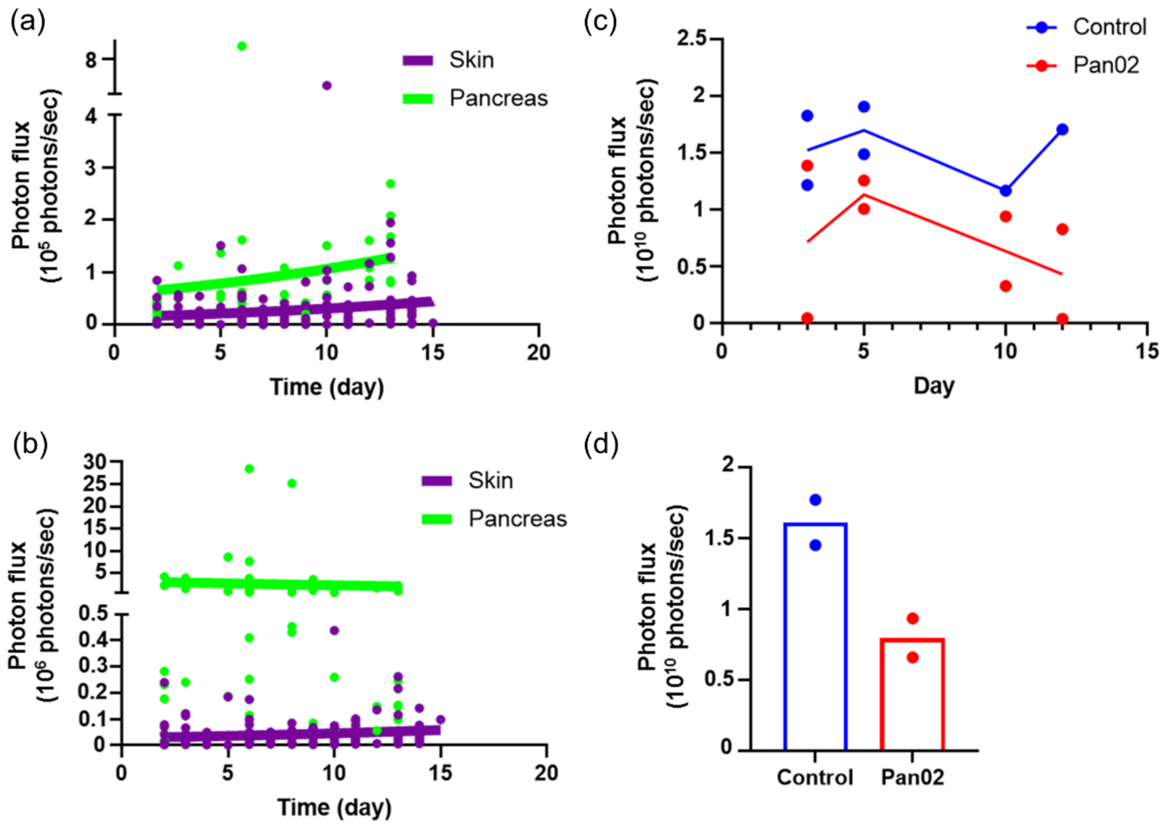

Figure S4. Bioluminescence quantification of (**a**) luminol and (**b**) L-012 signal comparing skin (n = 33, purple) and pancreas (n = 11, green) window chambers. Pancreas window chamber bioluminescent quantification of (**c**) p21 as a function of time, (**d**) time average of mean p21 activation in p21-FLuc pancreas window chamber-bearing animals without (blue) and with Pan02 Dendra2 reporter cells (red); n = 2 animals.

1.2. Image J Macro Code

```
//this runs as a sub macro to Fileprepandroi.ijm
//it's job is to measure and save results for each modality.
//it should be passed the modality then a comma then the file name
//this assumes that there is at least one old image open for the get director to function
// v 1.1 remove the measurements and save as analyze format

args = split(getArgument(),",");
name = args[2];
acqtime = args[1];

imagedir = getDirectory("image");
roiManager("Deselect");
roiManager("Delete");//start with clean roiset
run("Clear Results");//start with clean results

animalnumberstart = indexOf(name,"S")+1;
animalnumberend = indexOf(name," ");
```

```
snumber = substring(name,animalnumberstart,animalnumberend);

daystart = indexOf(name,"day")+4;
dayend = indexOf(name," ",daystart);
days = substring(name,daystart,dayend);

modality = args[0];

fmodalitystart = dayend+1;
fmodalityend = indexOf(name," ",fmodalitystart);
fluorescencemodality = substring(name,fmodalitystart,fmodalityend);

//build new photo for new functional image
String.resetBuffer;
String.append(imagedir);
String.append("S");
String.append(snumber);
String.append(" day ");
String.append(days);
String.append(" ");
String.append(modality);
String.append(" photo");
String.append(".tif");
newphotfilename = String.buffer();
String.resetBuffer;
open(newphotfilename);

run("In [+]");
run("In [+]");
run("In [+]");
run("In [+]");
run("In [+]");
setTool("angle");
waitForUser("Use the angle tool to draw ROI from the two bolts to the vertical then click ok")
run("Measure");

measureangle= getResult("Angle",0);

run("Specify...", "width=22 height=22 x=51 y=50 oval");

waitForUser("Move the oval to the window and click ok");
run("ROI Manager...");
roiManager("Add");
roiManager("select",0);
```

```
//build name for window roi;
String.resetBuffer;
String.append(imagedir);
String.append("S");
String.append(snumber);
String.append(" day ");
String.append(days);
String.append(" ");
String.append(modality);
String.append(".zip");
windowroi = String.buffer();
String.resetBuffer;
//end build name for window roi

roiManager("Rename",windowroi);
roiManager("select",0);
roiManager("Save Selected",call("ij.plugin.frame.RoiManager.getName", 0));


//build name for opening functional image;
String.resetBuffer;
String.append(imagedir);
String.append("S");
String.append(snumber);
String.append(" day ");
String.append(days);
String.append(" ");
String.append(modality);
String.append(".tif");
functionalfilename = String.buffer();
String.resetBuffer;
//end build name for functional image

open(functionalfilename);

roiManager("Select", 0);
run("Crop");

run("Clear Outside");

//build WC only filename
String.resetBuffer;
String.append(imagedir);
```

```
String.append("S");
String.append(snumber);
String.append(" day ");
String.append(days);
String.append(" ");
String.append(modality);
String.append(" WC only");
String.append(".tif");
WCfilename = String.buffer();
String.resetBuffer;
//end build name for WC

//saveAs("Tiff", WCfilename);

run("Divide...", "value=&acqtime");

//build norm filename
String.resetBuffer;
String.append(imagedir);
String.append("S");
String.append(snumber);
String.append(" day ");
String.append(days);
String.append(" ");
String.append(modality);
String.append(" WC only");
String.append(" norm_");

//get seconds before decimal
textacqtime = d2s(acqtime,2);
timearray = split(textacqtime,".");
secondsnumber = timearray[0];
//get seconds after decimal
restnumber = timearray[1];
//creat a file friendy number with p in place of decimal
newseconds = secondsnumber+"p"+restnumber;

String.append(newseconds);
String.append(".tif");
WCnormfilename = String.buffer();
String.resetBuffer;
//end build norm filename

//saveAs("Tiff",WCnormfilename);
```

```
run("Rotate... ", "angle=&measureangle grid=1 interpolation=Bicubic");

//create new rotatefilename
String.resetBuffer;
oldfilenamearray = split(WCnormfilename,".");
oldfilename = oldfilenamearray[0];
String.append(oldfilename);
String.append(" rotate");
//String.append(".tif");
WCnormrotfilename = String.buffer();
String.resetBuffer;
//end old rotatefilename

//saveAs("Tiff",WCnormrotfilename);

run("Analyze 7.5...","save=&WCnormrotfilename");
```
